# Expression of Mutant Huntingtin in Leptin Receptor-Expressing Neurons Does Not Control the Metabolic and Psychiatric Phenotype of the BACHD Mouse

**DOI:** 10.1371/journal.pone.0051168

**Published:** 2012-12-10

**Authors:** Sofia Hult Lundh, Rana Soylu, Åsa Petersén

**Affiliations:** Translational Neuroendocrine Research Unit, Department of Experimental Medical Science, Lund University, Lund, Sweden; Ohio State University, United States of America

## Abstract

Metabolic and psychiatric disturbances occur early on in the clinical manifestation of Huntington’s disease (HD), a neurodegenerative disorder caused by an expanded CAG repeat in the *huntingtin* (*HTT*) gene. Hypothalamus has emerged as an important site of pathology and alterations in this area and its neuroendocrine circuits may play a role in causing early non-motor symptoms and signs in HD. Leptin is a hormone that controls energy homeostasis by signaling through leptin receptors in the hypothalamus. Disturbed leptin action is implicated in both obesity and depression and altered circulating levels of leptin have been reported in both clinical HD and rodent models of the disease. Pathological leptin signaling may therefore be involved in causing the metabolic and psychiatric disturbances of HD. Here we tested the hypothesis that expression of mutant HTT in leptin receptor carrying neurons plays a role in the development of the non-motor phenotype in the BACHD mouse model. Our results show that inactivation of mutant HTT in leptin receptor-expressing neurons in the BACHD mouse using cross-breeding based on a cre-loxP system did not have an effect on the metabolic phenotype or anxiety-like behavior. The data suggest that mutant HTT disrupts critical hypothalamic pathways by other mechanisms than interfering with intracellular leptin signaling.

## Introduction

Huntington’s disease (HD) is a fatal neurodegenerative disorder caused by a CAG repeat expansion in the *huntingtin* (*HTT*) gene [1]. The classical triad of symptoms includes motor impairments, such as chorea and dystonia, cognitive decline and psychiatric disturbances [2], [3]. Clinical diagnosis currently requires onset of motor symptoms, which are associated with progressive and severe basal ganglia pathology [2]. It is now well known that psychiatric and cognitive disturbances as well altered sleep and metabolism often begin several years before motor onset but the underlying neuropathological mechanisms behind these symptoms are still not known [4], [5], [6], [7], [8], [9], [10]. Studies aiming to elucidate the pathogenic steps leading up to the non-motor phenotype in HD will be important both for the understanding of the disease and may also unravel targets for early disease modifying therapies for this disorder without a cure. Since hypothalamus is the main regulator of metabolism, sleep and emotions, pathological alterations in this area and neuroendocrine circuits may play a role in causing these early non-motor symptoms and signs in HD. Several hypothalamic and neuroendocrine changes have recently been identified in both clinical HD and in different rodent models of the disease [11], [12]. Altered levels of the metabolic hormone leptin have been reported in both clinical HD and rodent models of HD [6], [13], [14], [15]. Leptin is a peptide secreted into the blood by adipose tissue and that enters the brain via a saturable transport mechanism. It exerts its control of energy homeostasis by binding to leptin receptors in the hypothalamus, but leptin receptors are also expressed in other brain areas implicated in mood and emotion, such as the amygdala, hippocampus and cortex [16], [17], [18]. In fact, impaired leptin action in the central nervous system (CNS) has been implicated in both obesity and depression and a potential link between these conditions have been suggested [16], [19], [20].

The BACHD model is a transgenic mouse expressing full-length mutant HTT and that displays a metabolic phenotype and psychiatric-like behavior [15], [21], [22], [23]. It is constructed with LoxP sites flanking exon1 of mutant HTT, which makes it a powerful tool to investigate the effect of inactivation of mutant HTT in specific tissues and cells on behavior using cre-recombinase (cre) [21]. We have recently shown that the increased body weight with insulin and leptin resistance in the BACHD mouse could be prevented by expression of cre in hypothalamic neurons using adeno-associated viral vectors, indicating that mutant HTT actions in the hypothalamus drives the metabolic phenotype in these mice [15]. The hypothalamus consists of a number of interconnected nuclei with differential expression of neuropeptides and neuroendocrine receptors. In order to dissect out what neuronal population in the hypothalamus would be critical for this effect, we here tested the hypothesis that mutant HTT in the leptin receptor carrying neuronal subpopulation in the hypothalamus would be responsible for the metabolic phenotype as well as for the psychiatric-like disturbances in the BACHD mouse. Hence, the aim of this study was to investigate the impact of inactivation of mutant HTT in leptin receptor-expressing neurons in offspring from BACHD mice crossed with mice expressing cre after the leptin receptor gene (B6.129-Lepr^tm2(cre)Rck^/J,) [21], [24]. Our results suggest that mutant HTT causes metabolic and psychiatric-like disturbances in the BACHD mouse by other mechanisms than interfering with intracellular leptin signalling in the hypothalamus.

## Materials and Methods

### Ethics statement

All experimental procedures were approved by the Regional Ethical Committees in Lund, Sweden (Permit Number: M20-11).

### Animals

The BACHD mouse was produced with the bacterial artificial chromosome-mediated transgenic approach and expresses full-length mutant HTT containing 97 polyglutamine repeats [21]. BACHD mice were obtained from the Jackson Laboratories ((FVB/N strain; Bar Harbor, Maine). B6.129-Lepr^tm2(cre)Rck^/J mice (here abbreviated LepR-cre mice) express cre immediately 3′ of the stop codon in the last exon of the gene (DeFalco et al., 2001) and were obtained from the Jackson Laboratories (C57BL/6 strain). LepR-cre mice have no overt phenotype. We crossed the BACHD mice with the LepR-cre mice using either a male BACHD with a female LepR-cre mice or the reverse in order to investigate parental origin of effects in this setting. Only mice from the F1 generation were used in the experiments. The genotype was determined from tail samples using polymerase chain reaction (PCR) as described previously [21], [24]. Mice were followed until 6 months of age and were assessed using a combination of metabolic and behavioral readouts as described below. The number of mice from different genotypes and breedings is showed in Table S1. All mice were housed in groups with *ad libitum* access to normal chow diet and water, and were maintained at a 12 h light/dark cycle. B6.129X1-Gt(ROSA)26Sortm1(EYFP)Cos/J (here abbreviated ROSA-eYFP) mice were used to visualize activation of cre when bred with LepR-cre mice as they are produced with a *loxP*-flanked STOP sequence followed by the enhanced yellow fluorescent protein gene (EYFP) inserted into the *Gt(ROSA)26Sor* locus (C57BL/6 strain; Jackson laboratories).

### PCR for Cre-excision Validation

The successful deletion of HTT exon 1 in BACHD/LepR-cre was validated in genomic DNA from dissected fresh frozen brain regions at 6 months of age. DNA was extracted from the tissues with the DNeasy Blood and Tissue kit (Qiagen) and PCR was performed on a P×2 Thermal Cycler (Thermo Electron Corporation). The PCR products were run on a 1% agarose gel with SYBR® Safe DNA gel stain (Invitrogen). The primers were designed to detect the loxP flanked HTT exon 1 of the BACHD mice. Forward primer 5′-ATTCATTGCCCCGGTGCTGA-3′ and reverse primer 5′-AGCCCTCTTCCCTCTCAGACTAGAAGAGG-3′.

### Analysis of Leptin Receptor mRNA Expression

The mRNA expression of all isoforms of the leptin receptor (LepR) as well as the long form of the LepR was assessed in hypothalami from 2 months old male mice (n = 3–5/genotype). Mice were decapitated and the hypothalamus was dissected on a cold plate and then immediately frozen on dry ice until further stored at −80°C. mRNA expression analysis for all genotypes was carried out using quantitative real-time PCR (qRT-PCR). Total RNA was isolated by using RNeasy Lipid Tissue Kit (Qiagen) with an on-column DNase digestion (RNase-free DNase set, Qiagen) and cDNA was generated using random primers and SuperScript III Reverse Transcriptase (Invitrogen) according to the manufacturer’s instructions. SYBR Green I Master (Roche) was used for qRT-PCR and performed on LightCycler 480 (Roche) in a two-step cycling protocol. Calculations were performed by using the ΔΔCT method. Once normalized to β-actin, Hypoxanthine-guanine phosphoribosyltransferase (HPRT) and Glyceraldehyde 3-phosphate dehydrogenase (GAPDH) expression, the averaged expression value was estimated. The primer sequences used for the gene expressions are as followed: β-actin forward 5′-GCTGTGCTATGTTGCTCTA-3′, β-actin reverse 5′-TCGTTGCCAATAGTGATGA-3′, HPRT forward 5′-AGGAGAGAAAGATGTGATTGAT-3′, HPRT reverse 5′- TTAGATGCTGTTACTGATAGGAA-3′, GAPDH forward 5′- AACCTGCCAAGTATGATGA-3′, GAPDH reverse 5′-GGAGTTGCTGTTGAAGTC-3′, LepR-all isoforms forward 5′- AACTCAACTACGCTCTTCTG-3′, LepR-all isoforms reverse 5′- CCATCATCTGTGACTTCCAT-3′, LepR-long isoform forward 5′- AGTCACAAGATAATGGAGAATAAG-3′, LepR-long isoform reverse 5′- CTCTACTGGAATGGAACCTT-3′.

### Immunohistochemistry

Mice were anesthetized at 6 months of age with sodium pentobarbital (Apoteksbolaget) and transcardially perfused with ice-cold 4% paraformaldehyde (PFA). The brains were then removed and postfixed in PFA for 6 hours before cryoprotection in 25% sucrose. Coronal sectioning was made throughout the brain at 35 µm in 6 series at a freezing microtome and the sections were stored in an anti-freeze solution at −20°C until further processing. Free-floating brain sections were processed for green fluorescent protein (GFP; crossreacts with YFP) immunohistochemistry with a primary antibody against GFP (1∶20000, made in rabbit, Abcam). The sections were then incubated with a biotinylated secondary antibody (goat anti-rabbit IgG, 1∶200, Vector Laboratories) followed by an avidin-biotin peroxidase solution (ABC Elite, Vector Laboratories). Finally, the staining was visualized with 3,3′-diaminobenzidine (DAB).

### Metabolic Analyses

Body weight was assessed in all animals at 2 and 6 months of age. Body fat content was measured with a whole body dual energy x-ray absorptiometry (DEXA) scanner (Lunar PIXImus2, Lunar Corporation, Madison, WI, USA) during isofluran anesthesia at 6 months of age. Percentage of body fat was calculated with the PIXImus2 2.10 software (Lunar Corporation, Madison, WI, USA). Serum levels of leptin and insulin were assessed in duplicates with ELISA (Millipore and Crystal Chem Inc., respectively) according to the manufacturer’s instructions. Samples were diluted and re-run if needed.

### Behavioral Analyses

Anxiety- and depressive- like behavior were assessed in the elevated plus maze (EPM) and Porsolt forced swim test (FST), respectively, at 6 months of age. All tests were performed during the light phase of the circadian rhythm. Anxiety-like behavior was assessed in the EPM using the Ethovision 3.1 Software system (Noldus Information Technology). The EPM was elevated 50 cm above its base and consisted of four 30 cm long and 6 cm wide arms. Two of the arms were enclosed by 30 cm high walls. The mice were placed in the center of the maze and percentage time spent on the open arms during 5 min was calculated with the software.

Depressive-like behavior was assessed in the FST. The mice were placed in a 17 cm wide and 18 cm high glass cylinder filled with 10–12 cm of 25°C water. The mice were filmed for 6 min with a digital video camera and then wiped dry with a cloth. Total time spent immobile during the last 4 min of the test was estimated and calculated manually after the test. The criteria for immobility were that no swimming movements were made and that the movements made were only necessary to keep the head above the water surface according to the criteria defined by Porsolt 1977 [25]. Each mouse was given a random number so that the observer was blinded to its identity.

### Statistical Analyses

Data are presented as mean ± SEM. Statistical analyses were performed using PASW 19 statistical package (SPSS Inc. Chicago, Il). The data was analyzed with full factorial ANOVAs followed by appropriate *post hoc* tests. Significant statistical difference is considered at p<0.05. For detailed statistics see Statistical Results S1.

## Results

### Crossbreeding between BACHD and LepR-cre Mice

We have previously shown that BACHD mice develop increased body weight with leptin resistance [15]. BACHD mice were here crossbred with LepR-cre mice in order to inactivate mutant HTT in leptin receptor expressing neurons in the offspring in order to assess its effect on the metabolic and behavioral phenotype. We used two different breeding designs of the BACHD/LepR-cre mice since the body weight of both the mother and father may influence the metabolic phenotype of the offspring [26], [27]. In the first breeding we used a BACHD female and a LepR-cre male and in the second breeding we used a LepR-cre female and a BACHD male, and included only mice from the F1 generation in the experiment. LepR mice have normal body weight whereas BACHD mice have increased body weight typically from 2 months of age [15], [21]. Furthermore, leptin levels were found to be significantly elevated already at 2 months in female BACHD mice (4.50±0.45 ng/ml; n = 9) compared to WT littermates (2.77±0.49 ng/ml; n = 9; Student’s unpaired t-test, p = 0.019). At this time point, no differences in relative mRNA expression levels of LepR in the hypothalamus could be detected between any of the four genotypes. This was true for mRNA for all isoforms of the receptor (WT: 1.03±0.06, LepR-cre: 1.04±0.12, BACHD: 1.18±0.16, BACHD/LepR-cre: 1.23±0.13; 1-way ANOVA n.s.) and the long active isoform of the receptor (WT: 0.97±0.30, LepR-cre: 0.41±0.06, BACHD: 0.89±0.15, BACHD/LepR-cre: 0.42±0.05; 1-way ANOVA n.s.).

**Figure 1:**
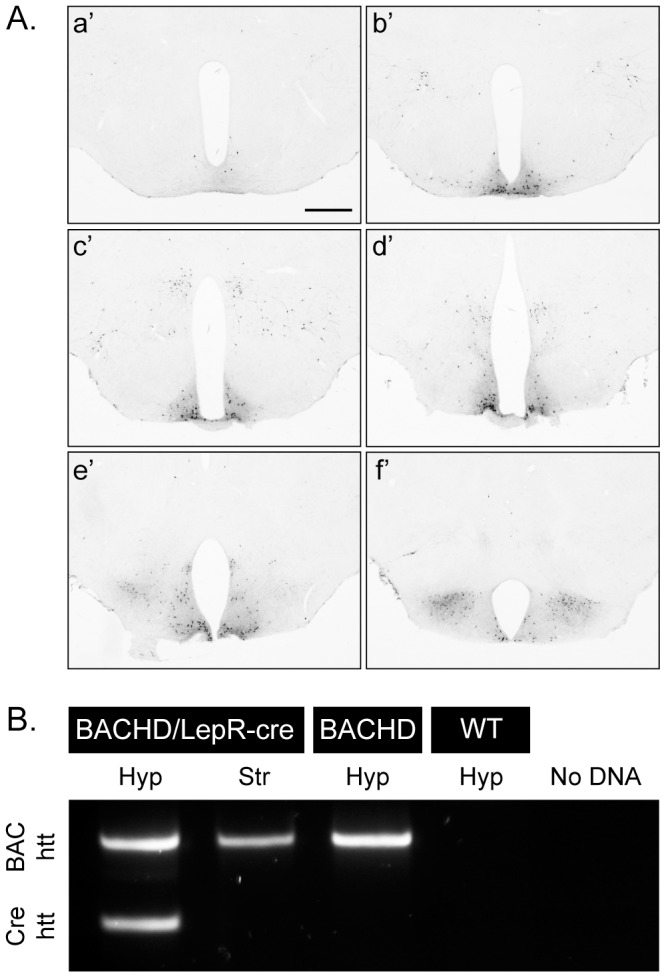
Demonstration and validation of Cre-excision. (A) GFP staining of hypothalamic sections from crossbred LepR-cre mice×ROSA-eYFP mice illustrates the cre-excision pattern in the hypothalamus, a’f’ represents rostral (bregma −1) to caudal (bregma −2.5) [51] hypothalamic sections, scale bar = 500 µm. (B) PCR analysis confirmed successful excision of human mutant htt exon1 in leptin receptor-expressing neurons in the hypothalamus, but not in a region (striatum), which lacks leptin receptor-expressing neurons.

We crossed M LepR-cre mice with F ROSA-eYFP mice in order to visualize the cre-excision pattern in the hypothalamus. Cre activity could be demonstrated in the arcuate, dorsomedial, ventromedial and lateral nuclei of hypothalamus, which is in line with previous studies (Figure 1A) [17]. In order to confirm that expression of mutant HTT was inactivated in the hypothalamus in BACHD/LepR-cre mice, PCR-analyses were performed to detect the excised product (Figure 1B).

### Inactivation of Mutant HTT in Leptin Receptor-expressing Neurons had no Effect on the Development of Metabolic Disturbances in BACHD Mice

**Figure 2:**
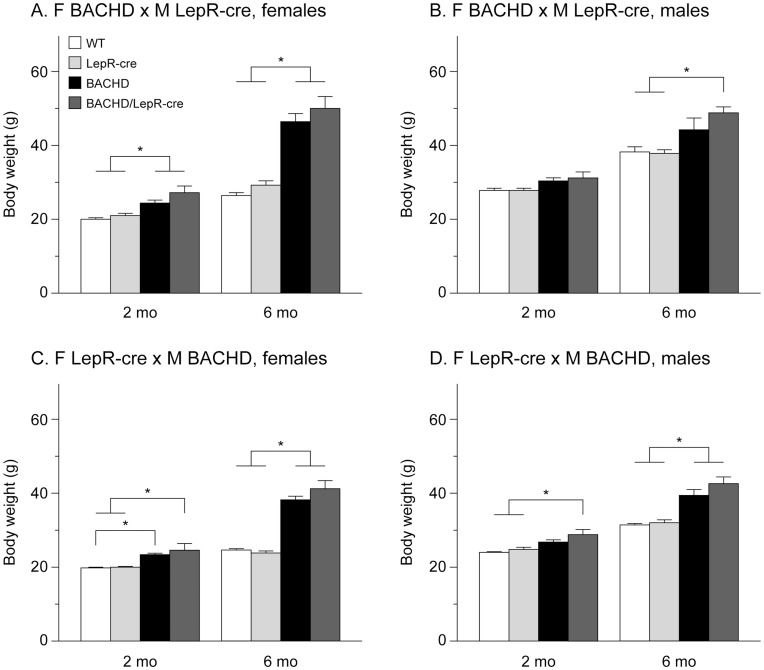
Body weight in BACHD×LepR-cre offspring. Body weight at 2- and 6-months in males and females from the two different breedings between BACHD and BACHD/LepR-cre (n = 4–10/genotype/sex/breeding). Both BACHD and BACHD/LepR-cre developed early onset obesity but no significant differences between BACHD and BACHD/LepR-cre could be detected in any of the breedings or sexes. (A) F BACHD×M LepR-cre, females, (B) F BACHD×M LepR-cre, males, (C) F LepR-cre×M BACHD, females, and (D) F LepR-cre×M BACHD, males. All data are expressed as means ± SEM. Significant differences from WT and/or LepR-cre mice: * p<0.05 (see Statistical Results S1).

**Figure 3:**
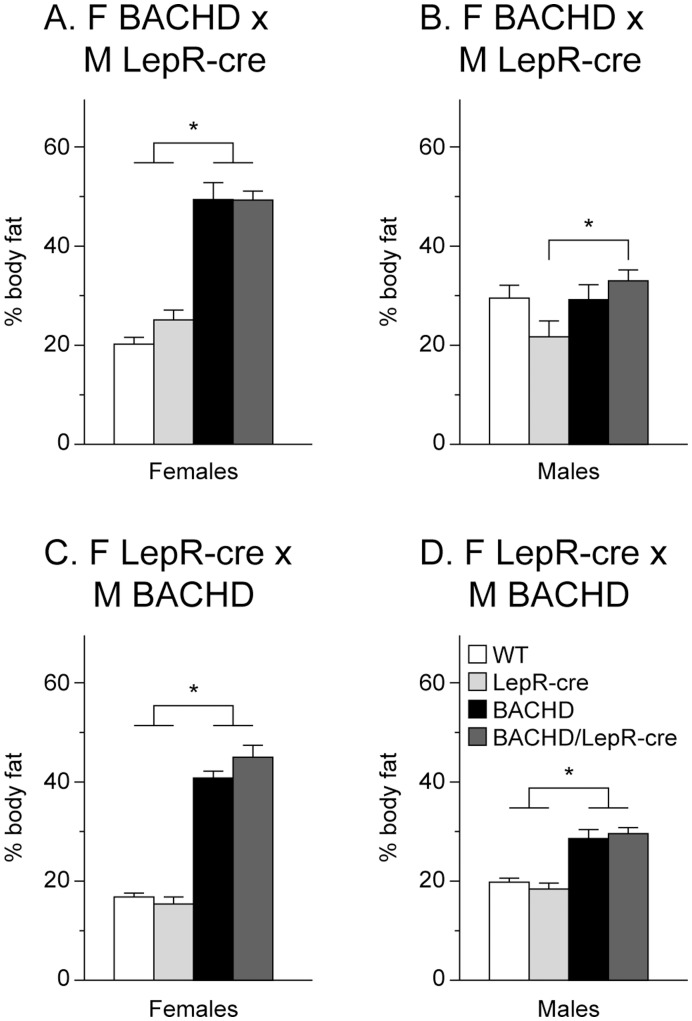
Body composition in BACHD×LepR-cre offspring. The graphs show percentage body fat as assessed with dual energy x-ray absorptiometry (DEXA) at 6-months in males and females from the two different breedings (n = 4–10/genotype/sex/breeding). Both BACHD and BACHD/LepR-cre showed increased percentage of body fat but there were no significant differences between BACHD and BACHD/LepR-cre in the two breedings and sexes. (A) F BACHD×M LepR-cre, females, (B) F BACHD×M LepR-cre, males, (C) F LepR-cre×M BACHD, females, and (D) F LepR-cre×M BACHD, males. All data are expressed as means ± SEM. Significant differences from WT and/or LepR-cre mice: * p<0.05 (see Statistical Results S1).

**Figure 4:**
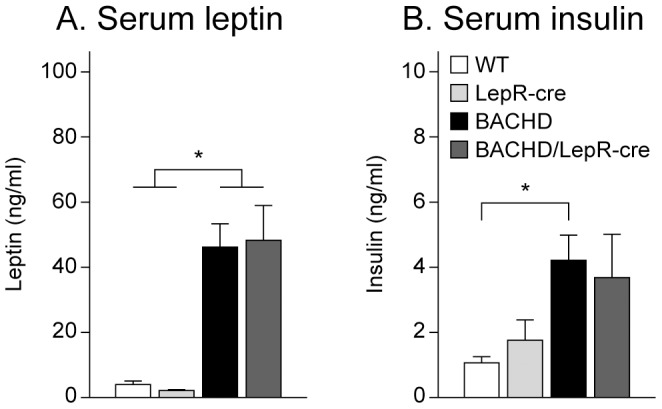
Endocrine measurements in BACHD×LepR-cre offspring. Circulating levels of leptin and insulin were measured in 6-month old female mice from the second breeding (n = 9–10/genotype). (A) Both BACHD and BACHD/LepR-cre showed elevated leptin levels but no significant differences between BACHD and BACHD/LepR-cre could be detected. (B) Only BACHD mice displayed significantly higher insulin levels than wt mice. However, no significant differences could be detected between BACHD and BACHD/LepR-cre. All data are expressed as means ± SEM. Significant differences from WT and/or LepR-cre mice: * p<0.05 (see Statistical Results S1).

Body weight was measured at 2 and 6 months of age. Both BACHD and BACHD/LepR-cre gained weight compared to the non-BACHD mice but we found no significant differences in body weight at any age between BACHD mice and BACHD/LepR-cre mice in any of the breedings. However, there were significant differences between the breedings, where offspring from a BACHD mother showed higher body weight than offspring from a LepR-cre mother (3-factor ANOVA (Figure 2). Accumulation of body fat was assessed with DEXA at 6 months of age. Both female and male BACHD mice and BACHD/LepR-cre in the two breedings displayed significantly higher percentage body fat than the other two groups mice (Figure 3). Again, a significant difference between the breedings could be detected, with higher body fat in offspring from a BACHD mother. To assess a potential effect on reported endocrine abnormalities of BACHD mice we measured serum levels of leptin and insulin in 6-month old female mice from the second breeding (F LepR-cre×M BACHD) [15]. Both BACHD and BACHD/LepR-cre mice displayed significantly elevated levels of circulating leptin compared to WT and LepR-cre mice (Figure 4A). For insulin, only BACHD mice displayed significantly higher levels than WT mice (Figure 4B). However, no differences in insulin levels could be detected between BACHD and BACHD/LepR-cre.

### No Effect on Development of Psychiatric Disturbances in the BACHD Mice when Inactivating Mutant HTT in Leptin Receptor-expressing Neurons

**Figure 5:**
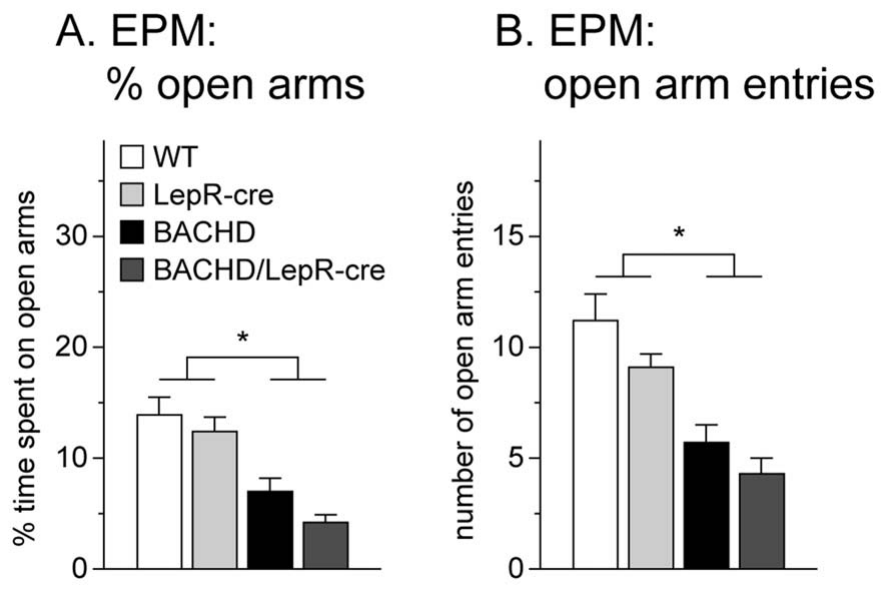
Assessment of anxiety-like behavior in BACHD×LepR-cre offspring. Both BACHD and BACHD/LepR-Cre displayed anxiety-like behavior at 6 months of age, with reduced time spent on open arms (A) as well as reduced number of entries onto the open arms (B) in the EPM at 6-months (n = 4–10/genotype/sex/breeding). However, no difference could be detected between BACHD and BACHD/LepR-cre mice. All data are expressed as means ± SEM. Significant differences from WT and LepR-cre mice: * p<0.05 (see Statistical Results S1).

Next, we sought to determine whether mutant HTT acting in leptin receptor-expressing neurons play any role in the development of psychiatric disturbances in BACHD mice. We assessed anxiety-like behavior in the EPM. This test is based on the natural conflict in rodents between the curiosity to explore a new environment and the tendency to avoid an open and potentially dangerous area. Less activity on the open arms is considered a measurement of anxiety-like behavior [28], [29]. We found no significant difference in time spent on open arms or in number of open arm entries in the EPM between 6 months old BACHD mice and BACHD/LepR-cre mice, but these two groups displayed significantly increased anxiety-like behavior compared to the control groups (Figure 5). There were no general differences between breedings or sexes and the data were therefore pooled.

**Figure 6:**
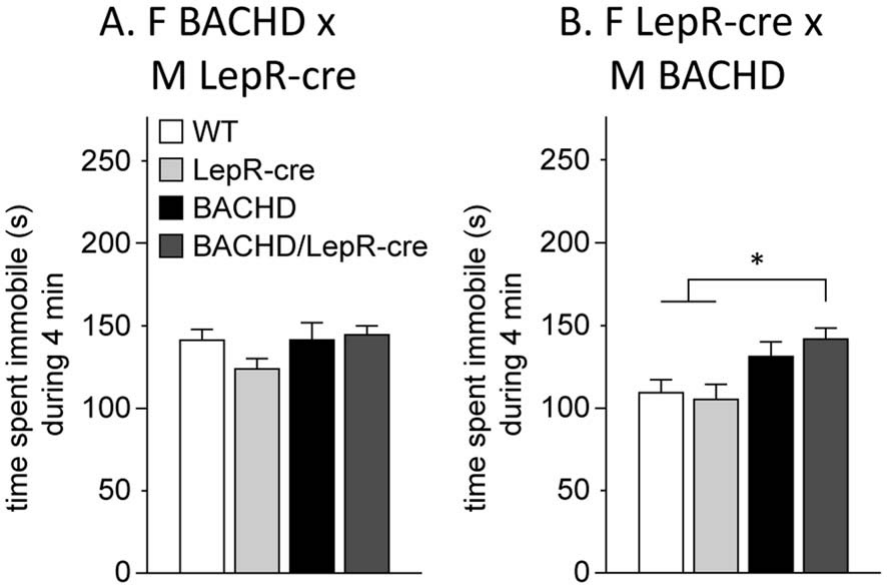
Assessment of depressive-like behavior in BACHD×LepR-cre offspring. No differences in immobility could be detected in the FST between any of the genotypes in the breeding between F BACHD and M LepR-cre (A). For the F LepR-cre×M BACHD breeding (B), LepR-cre mice spent significantly more time immobile than WT and LepR-cre mice. (n = 4–10/genotype/sex/breeding). All data are expressed as means ± SEM. significant differences from WT and LepR-cre mice: * p<0.05 (see Statistical Results S1).

We also studied depressive-like behavior in the FST in mice at 6 months of age. The FST is the most commonly used test for assessment of depressive-like behavior and screening for antidepressant drugs. In this test the mouse is put in a cylinder with water and after a while it may give up the struggle and just keep floating passively (immobile). This immobility is considered as a state of behavioral despair, a core symptom of depression, and can be decreased upon treatment with antidepressants [25]. We found significant differences between the breedings but no significant differences between the males and females, and data from both sexes were therefore pooled. For the first breeding between F BACHD and M LepR-cre we could not detect any differences in immobility between any of the genotypes (Figure 6A). For the second breeding between F LepR-cre and M BACHD, BACHD/LepR-cre mice spent more time immobile compared to WT and LepR-cre mice (Figure 6B). No significant differences in immobility were detected between BACHD and WT or LepR-cre mice indicating that these mice do not display depressive-like behavior. However, no differences were found between BACHD and BACHD/LepR-cre either, indicating that these two groups might display some depressive-like phenotype. In general, mice from all genotypes and breedings displayed high immobility (more than 2 minutes out of 4 minutes immobile). This could possibly be explained by a strain effect since the C57BL/6 mouse strain is known to show high immobility in this test [30]. The observed immobility likely reached a plateu and differences between the genotypes were therefore hard to detect.

## Discussion

The hypothalamus has emerged as an important site of pathology in HD but its relationship to non-motor symptoms and signs are still not fully understood. Defect leptin signaling has been implicated in the pathological process in both metabolic and psychiatric disturbances and altered levels of leptin have been reported in clinical HD [6], [14], [16], [20]. Interestingly, transgenic mouse models expressing full-length mutant HTT, the YAC128 and the BACHD mice, display an increase in body weight and body fat early on [15], [21], [31]. We have recently shown that the BACHD mouse develops leptin resistance and that inactivation of mutant HTT in the hypothalamus of young BACHD mice using administration of cre with AAV vectors could prevent the development of metabolic disturbances [15]. The viral vector approach in our previous experiment using a neuron specific promotor targeted around 120 000 (30%) of the hypothalamic neurons [15]. In order to dissect out what specific neuronal population in the hypothalamus would be responsible for the metabolic phenotype, we crossbred BACHD with LepR mice in order to inactivate mutant HTT in LepR containing neurons. However, we found that inactivation of mutant HTT selectively in LepR neurons did not have an effect on the development of weight gain, accumulation of body fat or increased leptin and insulin levels in BACHD mice. The data therefore suggest that leptin signaling in this model is still impaired and that the leptin resistance observed in BACHD is not caused by mutant HTT disrupting the intracellular leptin signaling in leptin receptor expressing neurons in the hypothalamus [15]. An estimation of the total number of leptin receptor-expressing neurons in hypothalamus based on the published literature gives a number of 10 000 in mice, hence inactivation o mutant HTT solely in this neuronal population is ten times less than in our previous AAV-cre paradigm targeting the hypothalamus [15], [17]. This indicates that in order to achieve an effect on the metabolic phenotype of the BACHD mouse, additional numbers and subtypes of hypothalamic neuronal populations need to be targeted with cre. Leptin receptor expressing neurons are found in several areas in the hypothalamus important for feeding regulation, such as the arcuate, dorsomedial, ventromedial and lateral hypothalamic (LH) nuclei. These nuclei host neurons that among others express proopiomelanocortin (POMC), agouti-related protein (AgRP), cocaine and amphetamine regulated transcript (CART), neuropeptide Y (NPY), melanin concentrating hormone (MCH) and orexin, all which are involved in the complex regulation of feeding behavior. Interestingly, MCH and orexin neurons in the LH have been reported to not co-express the leptin receptor, although leptin seem to regulate their action by innervation of these neurons by other leptin-receptor expressing neurons [32]. Hence, targeted inactivation of mutant HTT in specific hypothalamic neuronal populations involved in feeding regulation in the BACHD mouse model is a promising tool that will further address how mutant HTT disrupts and modulates these circuitries.

We performed two different breeding sets since it has been shown that both the mothers and fathers body weight might influence the metabolic phenotype of the offspring [26], [27]. We could observe significantly higher body weights and larger percentages of body fat in offspring that had an obese mother (BACHD) and lean father (LepR-cre) compared to offspring with a lean mother (LepR-cre) and an obese father (BACHD). Hence, in this experimental paradigm, the mother’s body weight had the largest influence on the body weight of the offspring. This may be an important factor to consider when designing experiments aiming at assessing metabolic dysfunction in BACHD mice and potentially also in other mouse models of the disease.

Circulating leptin levels have been analyzed in several animal models of HD and have been reported to be both increased and reduced depending on both the model used and the age of the animals. As shown in the present study, the full-length BACHD mouse displays increased leptin levels already from 2 months of age. Increased leptin levels have been reported in the other full-length HD model, the YAC128 mouse, from 12 months of age [33]. Rodent models expressing a fragment of the mutant HD gene display reduced leptin levels, in R6/2 from 6 weeks of age [13], the N171-82Q at its symptomatic phase [34] and the tgHD rat at 12 months of age [35]. However, the 140 CAG knock-in mouse model shows increased levels at 7 months of age and then decreased leptin levels at 22 months of age [13]. This finding together with the interpretation that the full-length mutant HTT models may represent an early phase of HD and the fragment mutant HTT models may mimic later stages, indicate a biphasic curve of leptin alterations. These could be mediated by hypothalamic dysfunction [15], albeit not from leptin receptor expressing neurons as indicated from the results in the present study. It is also possible that altered leptin levels in HD are a direct effect of the dysfunction of adipose tissue reported in HD mice [13], [36], [37].

Psychiatric disturbances such as depression and anxiety are common features of clinical HD [8], [9], [38]. Depressive-like features have been recapitulated in several mouse models of HD, both in shorter HTT fragment models, such as the R6/1 and N171-82Q as well as in the full-length models BACHD and YAC128 [23], [39], [40], [41], [42], [43], [44]. Also, anxiety-like behavior has been detected in the BACHD, the YAC128 and the 111 CAG knock-in mice [22], [43], [45]. The underlying biological mechanisms for the psychiatric aspects of HD are not fully known. Deficient leptin signaling in the brain has been hypothesized to link depression and metabolic dysregulation [16]. Leptin deficient mice display increased depressive and anxiety–related behaviors and overexpression of leptin has been shown to exert antidepressant and anxiolytic effects [20], [46], [47]. In clinical studies, leptin levels have been associated with both increased and reduced depressive symptoms [48], [49], [50]. As both patients with HD and mouse models of the disease display altered leptin levels, it would be possible that leptin could play a role in the development of psychiatric features in HD. When assessing anxiety-like behavior in the EPM in the mice in our study, we found that both BACHD and BACHD/LepR-cre spent less time and made fewer entries onto the open arms, indicative of an anxiety-like phenotype. This result is in agreement with findings by Menalled et al. where they showed that the BACHD mice display anxiety-like behavior in the light-dark choice test already at 3 months of age [22]. However, the BACHD/LepR-cre mice did not spent significantly more time than BACHD mice on the open arms indicating that inactivation of mutant HTT selectively in LepR neurons did not have an effect on the development of anxiety-like behavior in BACHD mice.

For assessment of depressive-like behavior we utilized the commonly used Porsolt forced swim test (FST). Mice from all genotypes and genders displayed high immobility in this test. Pouladi et al. demonstrated in a recent study that 12-month-old BACHD mice on the FVB/N background display depressive-like behavior in the same test [23]. The mice in our study are on a mixed background of FVB/N and C57BL/6. Mice of the FVB/N strain are known to show low immobility in the FST while the C57BL/6 strain is known to be one of the most immobile mouse strains [30]. One explanation to the observed high immobility in all genotypes in this study could therefore be a strain effect, as all genotypes appeared to reach a plateau of high immobility where potential differences would be hard to detect. It is therefore not possible to draw any definite conclusions regarding the effect of inactivation of mutant HTT expression in leptin receptor expressing neurons on depressive-like behavior in the BACHD mice from this experiment. Interestingly, BACHD/LepR-cre mice displayed significantly higher immobility than WT and LepR-cre mice in the second breeding (F LepR-cre×M BACHD). Also, BACHD mice in this breeding showed a trend of being more immobile than WT and LepR-cre mice. Since the BACHD mice were not significantly different from the BACHD/LepR-cre mice, it is possible that they do display a depressive-like phenotype. In light of this finding, it seems unlikely that inactivation of mutant HTT in leptin receptor expressing neurons have an effect on depressive-like behavior in this mouse model of HD. Although no sex dissimilarities could be detected in the FST, a significant difference between the two breedings was identified. This is an interesting finding that may reflect that there indeed are some sex specific differences, since offspring from a BACHD female show higher immobility than offspring from a BACHD male. Sex specific differences in terms of depressive-like behavior have been reported in the R6/1 mouse of HD, where female mice display a more depressive-like phenotype than male mice [39], [41]. It is also worth noting that the increase in immobility was accompanied by a higher body weight in offspring from a BACHD female. This further points to a potential link between obesity and depression.

In conclusion, the results from this study suggest that mutant HTT is not causing metabolic and psychiatric disturbances in the BACHD mouse model of HD by disrupting leptin-signaling downstream of the leptin receptor. We have recently shown that overexpression of mutant HTT in the hypothalamus leads to metabolic disturbances and that inactivation of mutant HTT in the hypothalamus of young BACHD mice can prevent the development of a metabolic phenotype [15]. The mechanisms by which the mutant protein disrupts hypothalamic pathways need to be further elucidated in order to identify the critical neuronal populations responsible for causing the metabolic phenotype.

## Supporting Information

Table S1 **Number of assessed mice.**
(DOCX)

Statistical results S1 **Statistical results.**
(DOCX)
