# Supplementary material for: Expression of Mutant Huntingtin in Leptin Receptor-Expressing Neurons Does Not Control the Metabolic and Psychiatric Phenotype of the BACHD Mouse
Source: PLoS One. 2012 Dec 10;7(12):e51168. doi: 10.1371/journal.pone.0051168 (PMC3519539; doi:10.1371/journal.pone.0051168)
Supplement: Table S1 — Number of assessed mice. (DOCX) [file pone.0051168.s001.docx]

**Table S1.** Number of assessed mice

|  | **WT** | | **LepR-cre** | | **BACHD** | | **BACHD/ LepR-cre** | |
| --- | --- | --- | --- | --- | --- | --- | --- | --- |
|  | **M** | **F** | **M** | **F** | **M** | **F** | **M** | **F** |
| **F BACHD x M LepR-cre** | 10 | 9 | 10 | 9-10 | 4 | 7 | 10 | 6 |
| **F LepR-cre x M BACHD** | 10 | 10 | 10 | 9-10 | 8 | 10 | 10 | 9 |

F=females, M=males
