# Supplementary material for: Expression of Mutant Huntingtin in Leptin Receptor-Expressing Neurons Does Not Control the Metabolic and Psychiatric Phenotype of the BACHD Mouse
Source: PLoS One. 2012 Dec 10;7(12):e51168. doi: 10.1371/journal.pone.0051168 (PMC3519539; doi:10.1371/journal.pone.0051168)
Supplement: Statistical results S1 — Statistical results. (DOCX) [file pone.0051168.s002.docx]

**Statistical Results S1**

Only significant p-values (<0.05) for interactions and *post hoc* tests are reported.

**Figure 2 (body weight):**

3-way ANOVA at 2 mo (all):

Breeding: F_(1,126)_=20.99, p<0.001

Sex: F_(1,126)_=117.51, p<0.001

Genotype: F_(3,126)_=26.20, p<0.001

Breeding*Sex: F_(1,126)_=4.46, p=0.037

3-way ANOVA at 6 mo (all):

Breeding: F_(1,126)_=67.65, p<0.001

Sex: F_(1,126)_=34.35, p<0.001

Genotype: F_(3,126)_=122.31, p<0.001

Breeding*Sex: F_(1,126)_=12.83, p<0.001

A) F BACHD x M LepR-cre, females:

1-way ANOVA at 2 mo:

F_(3,27)_=12.70, p<0.001

Tukey HSD *post hoc* at 2 mo:

BACHD vs. WT, p=0.007
BACHD vs. LepR-cre, p=0.045
BACHD/LepR-cre vs. WT, p<0.001
BACHD/LepR-cre vs. LepR-cre, p<0.001

1-way ANOVA at 6 mo:

F_(3,27)_=45.70, p<0.001

Tukey HSD *post hoc* at 6 mo:

BACHD vs. WT, p<0.001
BACHD vs. LepR-cre, p<0.001
BACHD/LepR-cre vs. WT, p<0.001
BACHD/LepR-cre vs. LepR-cre, p<0.001

B) F BACHD x M LepR-cre, males:

1-way ANOVA at 2 mo:

n.s. (F_(3,30)_=2.92, p=0.050)

1-way ANOVA at 6 mo:

F_(3,30)_=13.22, p<0.001

Tukey HSD *post hoc* at 6 mo:

BACHD/LepR-cre vs. WT, p<0.001
BACHD/LepR-cre vs. LepR-cre, p<0.001

C) F LepR-cre x M BACHD, females:

1-way ANOVA at 2 mo:

F_(3,35)_=7.32, p=0.001

Tukey HSD *post hoc* at 2 mo:

BACHD vs. WT, p=0.037
n.s. (BACHD vs. LepR-cre, p=0.057)
BACHD/LepR-cre vs. WT, p=0.003
BACHD/LepR-cre vs. LepR-cre, p=0.004

1-way ANOVA at 6 mo:

F_(3,35)_=54.09, p<0.001

Tukey HSD *post hoc* at 6 mo:

BACHD vs. WT, p<0.001
BACHD vs. LepR-cre, p<0.001
BACHD/LepR-cre vs. WT, p<0.001
BACHD/LepR-cre vs. LepR-cre, p<0.001

D) F LepR-cre x M BACHD, males:

1-way ANOVA at 2 mo:

F_(3,34)_=6.65, p=0.001

Tukey HSD *post hoc* at 2 mo:

BACHD/LepR-cre vs. WT, p=0.001
BACHD/LepR-cre vs. LepR-cre, p=0.009

1-way ANOVA at 6 mo:

F_(3,34)_=21.33, p<0.001

Tukey HSD *post hoc* at 6 mo:

BACHD vs. WT, p<0.001
BACHD vs. LepR-cre, p=0.001
BACHD/LepR-cre vs. WT, p<0.001
BACHD/LepR-cre vs. LepR-cre, p<0.001

**Figure 3 (body fat):**

3-way ANOVA:

Breeding: F_(1,127)_=27.75, p<0.001

Sex: F_(1,127)_=41.37, p<0.001

Genotype: F_(3,127)_=99.18, p<0.001

Sex*Genotype: F_(1,127)_=30.94, p<0.001

Breeding*Sex*Genotype: F_(3,127)_=2.69, p=0.049

A) F BACHD x M LepR-cre, females:

1-way ANOVA:

F_(3,28)_=49.97, p<0.001

Tukey HSD *post hoc*:

BACHD vs. WT, p<0.001
BACHD vs. LepR-cre, p<0.001
BACHD/LepR-cre vs. WT, p<0.001
BACHD/LepR-cre vs. LepR-cre, p<0.001

B) F BACHD x M LepR-cre, males:

1-way ANOVA:

F_(3,30)_=3.29, p=0.034

Tukey HSD *post hoc*:

BACHD/LepR-cre vs. LepR-cre, p=0.023

C) F LepR-cre x M BACHD, females:

1-way ANOVA:

F_(3,35)_=99.24, p<0.001

Tukey HSD *post hoc*:

BACHD vs. WT, p<0.001
BACHD vs. LepR-cre, p<0.001
BACHD/LepR-cre vs. WT, p<0.001
BACHD/LepR-cre vs. LepR-cre, p<0.001

D) F LepR-cre x M BACHD, males:

1-way ANOVA:

F_(3,34)_=21.89, p<0.001

Tukey HSD *post hoc*:

BACHD vs. WT, p<0.001
BACHD vs. LepR-cre, p<0.001
BACHD/LepR-cre vs. WT, p<0.001
BACHD/LepR-cre vs. LepR-cre, p<0.001

**Figure 4 (leptin and insulin):**

A) Leptin (F LepR-cre x M BACHD, females):

1-way ANOVA:

F_(3,35)_=17.23, p<0.001

Tukey HSD *post hoc*:

BACHD vs. WT, p<0.001
BACHD vs. LepR-cre, p<0.001
BACHD/LepR-cre vs. WT, p<0.001
BACHD/LepR-cre vs. LepR-cre, p<0.001

B) Insulin (F LepR-cre x M BACHD, females):

1-way ANOVA:

F_(3,34)_=3.53, p=0.025

Tukey HSD *post hoc*:

BACHD vs. WT, p=0.038

**Figure 5 (anxiety-like behavior):**

A) EPM: % time spent on open arms:

3-way ANOVA (all):

Breeding: n.s. (F_(1,127)_=0.19, p=0.661)

Sex: n.s. (F_(1,127)_=0.05, p=0.819)

Genotype: F_(3,127)_=12.90, p<0.001

Tukey HSD *post hoc*:

BACHD vs. WT, p=0.002
BACHD vs. LepR-cre, p=0.025
BACHD/LepR-cre vs. WT, p<0.001
BACHD/LepR-cre vs. LepR-cre, p<0.001

B) EPM: Open arm entries:

3-way ANOVA (all):

Breeding: n.s. (F_(1,127)_=0.14, p=0.707)

Sex: n.s. (F_(1,127)_=2.34, p=0.129)

Genotype: F_(3,127)_=13.57, p<0.001

Tukey HSD *post hoc*:

BACHD vs. WT, p<0.001
BACHD vs. LepR-cre, p=0.036
BACHD/LepR-cre vs. WT, p<0.001
BACHD/LepR-cre vs. LepR-cre, p=0.001

**Figure 6 (depressive-like behavior):**

3-way ANOVA (all):

Breeding: F_(1,124)_=7.30, p=0.008

Sex: n.s. (F_(1,124)_=3.87, p=0.051)

Genotype: F_(3,124)_=5.47, p=0.001

A) F BACHD x M LepR-cre:

1-way ANOVA:

n.s. (F_(3,62)_=2.09, p=0.110)

B) F LepR-cre x M BACHD:

1-way ANOVA:

F_(3,70)_=4.77, p=0.004

Tukey HSD *post hoc*:

BACHD/LepR-cre vs. WT, p=0.030
BACHD/LepR-cre vs. LepR-cre, p=0.009
